# HiDeS: a higher-order-derivative-supervised neural ordinary differential equation for multi-robot systems and opinion dynamics

**DOI:** 10.3389/fnbot.2024.1382305

**Published:** 2024-03-12

**Authors:** Meng Li, Wenyu Bian, Liangxiong Chen, Mei Liu

**Affiliations:** ^1^Zhangjiajie College, Zhangjiajie, China; ^2^School of Public Administration, Hunan University, Changsha, China

**Keywords:** neural ordinary differential equations, multi-robot systems, opinion dynamics, robotics, neural networks

## Abstract

This paper addresses the limitations of current neural ordinary differential equations (NODEs) in modeling and predicting complex dynamics by introducing a novel framework called higher-order-derivative-supervised (HiDeS) NODE. This method extends traditional NODE frameworks by incorporating higher-order derivatives and their interactions into the modeling process, thereby enabling the capture of intricate system behaviors. In addition, the HiDeS NODE employs both the state vector and its higher-order derivatives as supervised signals, which is different from conventional NODEs that utilize only the state vector as a supervised signal. This approach is designed to enhance the predicting capability of NODEs. Through extensive experiments in the complex fields of multi-robot systems and opinion dynamics, the HiDeS NODE demonstrates improved modeling and predicting capabilities over existing models. This research not only proposes an expressive and predictive framework for dynamic systems but also marks the first application of NODEs to the fields of multi-robot systems and opinion dynamics, suggesting broad potential for future interdisciplinary work. The code is available at https://github.com/MengLi-Thea/HiDeS-A-Higher-Order-Derivative-Supervised-Neural-Ordinary-Differential-Equation.

## 1 Introduction

As a learnable model parameterized by **θ** ∈ ℝ^*n*^, a standard neural ordinary differential equation (NODE) $\dot{x}=\phi_{\theta }\left(x,t\right)$ is particularly adept at representing complex and nonlinear dynamics (Chen et al., 2018; Liufu et al., 2024), where *x* ∈ ℝ^*d*^ is the state at time *t*, $\dot{x}=\text{d}x/\text{d}t$ denotes the time derivative of *x*, and **ϕ**(*x, t*) is a vector field with **ϕ** ∈ (ℝ^*d*^×ℝ → ℝ^*d*^) being a function of *x* and *t*. Its strength lies in processing time-variant data and adaptively learning from it. This modeling flexibility renders NODEs great potential for the intricate nature of dynamic systems (Hua et al., 2023; Wang et al., 2023; Jin et al., 2024), enabling a more nuanced understanding of complex dynamic systems.

Despite these strengths, the standard NODE encounters expressivity limitations, failing to model functions like NOT operations (Kidger, 2021; Xu et al., 2023). The NOT operation [i.e., (0, 1) → (1, 0)] involves trajectories that necessarily intersect, presenting a challenge for standard NODEs that cannot model intersecting trajectories due to their first-order nature. The NODE with momentum, which can be regarded as a second-order ODE, improves the expressive capability (Sander et al., 2021): $\ddot{x}=c_{0}\dot{x}+c_{1}\phi_{\theta }\left(x,t\right)$, where *c*_0_ and *c*_1_ are constants. Nonetheless, it can only express limited dynamics due to the linear relationship of $\dot{x}$ and **ϕ**_θ_(*x, t*). Besides, it cannot model interactions between $\dot{x}$ and *x*. The second-order NODE (SONODE) presented in Norcliffe et al. (2020) seeks to address this limitation by modeling the interactions between $\dot{x}$ and *x*. However, SONODE cannot model interactions between higher-order derivatives and *x*, and the supervised signal used in training is only the ground-truth value of *x*, which confines its scope and limits its prediction capability.

To surmount these challenges, we propose a higher-order-derivative-supervised NODE (HiDeS NODE) that is able to model interactions between higher-order derivatives and *x*. This approach not only expands the expressive range of NODEs but also enhances predictive ability through employing the state vector and its higher-order derivatives as supervised signals, surpassing the modeling and predicting performance of existing NODEs.

This paper evaluates the effectiveness of the HiDeS NODE in the realms of multi-robot systems and opinion dynamics, key areas of dynamic systems, both domains that inherently involve complex interactions and communication (Granha et al., 2022). In multi-robot systems, conventional analytic solutions fall short in high-dimensional control tasks (Károly et al., 2021), such as multi-robot grasping and motion control. NODEs, in contrast, offer a promising avenue for modeling and controlling complex dynamic interactions in a continuous, efficient, and adaptable manner in multi-robot systems. Regarding opinion dynamics research, the primary objective is to decipher the underlying mechanisms and influences that catalyze shifts in opinions. Existing methodologies for learning opinion dynamics overlook the critical prior knowledge that opinion dynamics can be described as an ODE formulated as $\dot{x}=\phi \left(x,t\right)$. ODEs are particularly well-suited for modeling the fluid nature of opinion dynamics due to their inherent capacity to capture the dynamics of evolving systems. However, contemporary models employed in learning opinion dynamics underutilize this foundational knowledge. This oversight hampers their ability to effectively capture the nuanced and intricate nature of opinion evolution. Furthermore, the complexities inherent in the evolution of opinions present considerable challenges to the application of existing NODEs in both modeling and forecasting the trajectories of opinion dynamics. The HiDeS NODE conquers these aspects, providing a more effective tool for understanding and predicting opinion evolution.

**Figure 1:**
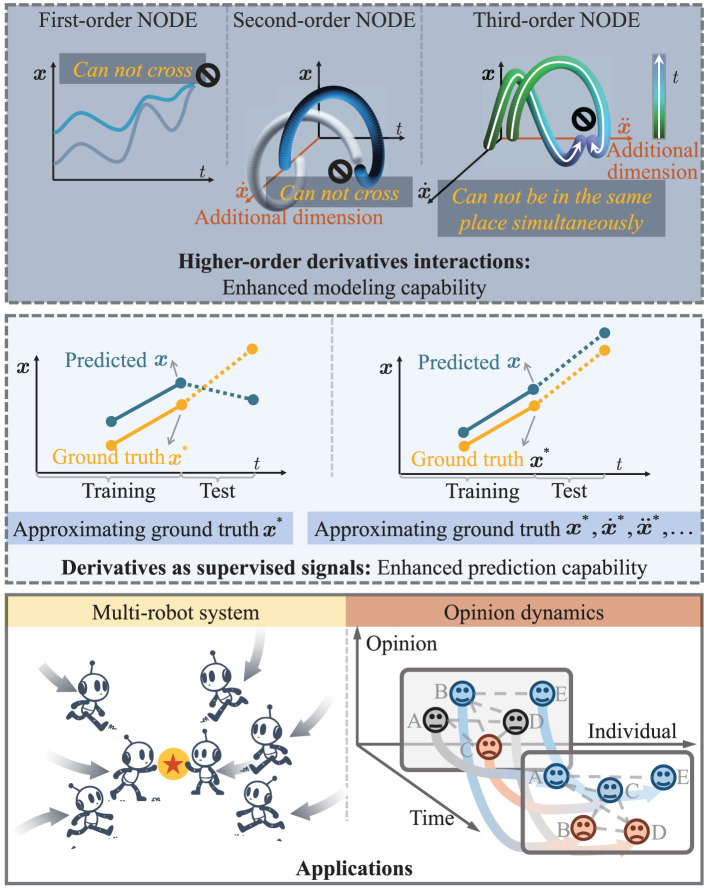
Framework of this paper. **(Top)** Evolution from first to third-order NODEs, highlighting their progressively sophisticated ability to model complex trajectories where higher orders allow for more intricate behaviors. **(Middle)** Predicting precision with and without using higher-order derivatives as supervised signals, showing the latter's superior approximation of ground truth. **(Bottom)** Practical applications of HiDeS NODEs in multi-robot systems and opinion dynamics.

To bridge these gaps, we propose a new NODE, termed HiDeS NODEs, for modeling and predicting tasks in multi-robot control and opinion dynamics. Figure 1 illustrates the framework of the HiDeS NODE, and Table 1 qualitatively demonstrates the HiDeS NODE's superiority compared with existing NODEs.

**Table 1:** Comparisons among different NODEs.

**Models**	**Year**	**Modeling higher-order dynamics**	**Modeling derivative interactions of each order**	**Applied to opinion dynamics**	**Supervised signals**
Standard NODE (Chen et al., 2018)	2018	×	×	×	*x*(*t*)
Momentum NODE (Sander et al., 2021)	2021	Only 2nd order	×	×	*x*(*t*)
SONODE (Norcliffe et al., 2020)	2020	Only 2nd order	√	×	*x*(*t*)
HiDeS NODEs	2024	√	√	√	$x\left(t\right),\dot{x}\left(t\right),\ldots ,x^{\left(c-1\right)}$

The contributions of this paper are demonstrated as follows:

- We propose the HiDeS NODE, a novel approach for modeling the intricacies of dynamics. The HiDeS NODE excels in modeling and predicting interactions among higher-order derivatives within dynamic systems. This advancement provides a more accurate and nuanced representation of dynamic systems.
- The HiDeS NODE integrates higher-order derivatives as supervised signals, significantly enhancing the ability to predict dynamical behaviors.
- We examine the versatility and effectiveness of the proposed HiDeS NODE through its application in two distinct yet complex fields: Multi-robot systems and opinion dynamics. In these fields, the model's ability to capture and predict intricate system dynamics is evaluated.
- To our knowledge, this is the first time that the NODE is introduced for opinion dynamics and multi-robot-system control. Application of the proposed HiDeS NODE to these fields unveils new avenues for both the advancement of NODE methodologies and the nuanced modeling of opinion dynamics and multi-robot-system control.

## 2 Related work

In this section, we briefly review three lines of research that are close to our work: NODEs, multi-robot-system control methods, and opinion dynamics modeling.

### 2.1 NODEs

The intersection of neural networks and differential equations, especially interpreting residual networks (ResNets) as discretized ODEs, spurs the development of NODEs (Weinan, 2017; Cui et al., 2023; Ruiz-Balet and Zuazua, 2023). NODEs integrate black-box ODE solvers and neural networks to parameterize the hidden state's derivative. This integration substantially advances time-series modeling, offering robust function approximation and handling of irregular data (Chen et al., 2018; Kidger, 2021). However, standard NODEs encounter representational constraints without dimensionality augmentation, constraining their universal approximation capabilities for certain functions (Dupont et al., 2019).

Research pivots toward higher-dimensional NODEs to overcome these limitations. Momentum-enhanced ResNets, representing second-order NODE extensions, exhibit enhanced capability in modeling non-homeomorphic dynamics and demonstrate improved convergence properties (Sander et al., 2021). In parallel, augmented NODEs, by expanding the solution space, facilitate the learning of more complex functions through simpler dynamic flows, thereby sidestepping the limitations of the vector field's general-representation property (Kidger, 2021). Nonetheless, augmented NODEs introduce challenges in interpretability and alter the loss landscape's structure (Norcliffe et al., 2020). A specific iteration of augmented NODEs, termed second-order NODEs (SONODEs) (Norcliffe et al., 2020), captures more intricate behaviors by integrating second-order dynamics, effectively combining the principles of coupled augmented NODEs. Additionally, the advent of heavy ball NODEs (HBNODEs) (Xia et al., 2021) marks a significant advancement. HBNODEs incorporate the classical momentum accelerated gradient descent method and adeptly mitigate the vanishing gradient problem, thereby enhancing the model's capacity in learning long-term dependencies in sequential data (Xia et al., 2021).

### 2.2 Multi-robot-system control

Multi-robot systems provide significant benefits in tasks that demand the duplication of effort, risk reduction, or adaptability, offering distinct advantages over single-robot systems (Hichri et al., 2022; Kwa et al., 2022). Multi-robot-system control methods can be categorized into deterministic methods with fixed forms and learning-based methods (Pierpaoli et al., 2021). However, deterministic methods lack flexibility and adaptability in dynamic or unpredictable environments (Liu et al., 2023). In order to overcome these defects, learning methods are increasingly applied to multi-robot control problems. Adaptation methods, for instance, are proposed to enhance trajectory prediction efficiency in multi-agent systems (Aydemir et al., 2023). Furthermore, the parameter-adaptive learning methods are improved through iterative parametric learning controllers (Yu and Chen, 2023). Additionally, neural network-based adaptive learning methods are utilized to learn unknown fault functions, ensuring cooperative tracking in distributed multi-robot systems (Khalili et al., 2020). Despite these advancements, existing methods often fall short in naturally and efficiently modeling the dynamics that often can be described as an ODE, a gap that NODEs can potentially fill.

### 2.3 Opinion dynamics modeling

Opinion dynamics studies how opinions form and evolve over time through interactions with individuals and environments. Researchers propose various mathematical models to understand and predict the dynamics of opinions. These include continuous-time models such as the DeGroot model (Wu et al., 2023), Hegselmann-Krause model, and bounded confidence model (Kolarijani et al., 2021), as well as discrete-time models like the Ising model, Voter model, and Friedkin and Johnsen model (Baumann et al., 2020; Ao and Jia, 2023; Peng et al., 2023). However, these models, with their fixed forms, lack the flexibility to model the evolution of opinions independently.

In response to these limitations, researchers leverage advances in neural networks to utilize their nonlinear relation approximation ability for learning complex opinion dynamics. An early approach introduces a linear influence model that learns edge influence strength from real data (De et al., 2014). Unlike traditional models, this linear model represents a foundational step in opinion dynamics learning methods, but its simplicity fails to capture the complexity of societal opinion dynamics. Furthering this exploration, SLANT (De et al., 2016; Zhu et al., 2020) introduces a linear model of latent opinions driven by stochastic differential equations (SDEs) using historical, fine-grained event data. Subsequently, SLANT+ (Kulkarni et al., 2017) extends this model with a nonlinear generative model and a network-guided recurrent neural network (RNN) architecture. This model underscores the importance of nonlinearity in designing opinion dynamics models. However, the RNN architecture it relies on faces the challenge of the vanishing gradient problem, hindering long-term predictions of opinion flow. Learnable opinion dynamics model (LODM) (Monti et al., 2020) emerges as a learnable generalization of an opinion dynamics model, combining the causal interpretability of traditional agent-based models with data-driven approaches. Additionally,Okawa and Iwata (2022) introduces the sociologically-informed neural network (SINN), a novel hybrid approach that integrates sociological and social psychological theories with data-driven neural networks to model and predict opinion dynamics in social networks. Despite these advances, current models do not fully exploit the prior knowledge of differential equations in opinion evolution, nor do they effectively model higher-order derivatives.

## 3 Materials and methods

In this section, formal descriptions and analyses of the proposed HiDeS NODE are provided. Table 2 presents the main symbols and notations used throughout this paper to ensure clarity and ease of understanding.

**Table 2:** Main symbols and notations.

**Symbol**	**Description**
*x*	State vector representing opinions of individuals
*t*	Time variable
$\dot{x}$	First-order time derivatives of *x*
$\ddot{x}$	Second-order time derivatives of *x*
$\overset{\mathrm{...}}{x}$	Third-order time derivatives of *x*
*c*	Order of the highest derivative in HiDeS NODE
*x* ^(*c*)^	*c*-th order time derivative of *x*
θ	Parameters of a neural network
ϕ_θ_(·)	Vector field (neural network) parameterized by θ
ℝ^*d*^	*d*-dimensional real space
ℝ^*n*^	*n*-dimensional real space
ω	Extended state vector in HiDeS NODE
Δ*t*	Time step for numerical approximation
ϑ	Alternate set of parameters for the neural network
$\overset{\vee }{t},\hat{t}$	Time interval boundaries

### 3.1 Formulation of the HiDeS NODE

The HiDeS NODE has two unique features for modeling and predicting opinion evolution. The first is that the HiDeS NODE is a higher-order NODE that is able to model interactions of higher-order derivatives of the opinion variable *x*. The second is that the HiDeS NODE adopts higher-order derivatives as supervisory signals to predict opinion evolution better. The HiDeS NODE is described as Equation (1):


(1) $$\left[\begin{array}{c} \dot{x}(t)\\ \ddot{x}(t)\\ \overset{...}{x}(t)\\ \vdots \\ x^{\left(c\right)}(t) \end{array}\right]=\phi_{\theta }\left(\left[\begin{array}{c} x(t)\\ \dot{x}(t)\\ \ddot{x}(t)\\ \vdots \\ x^{\left(c-1\right)}(t) \end{array}\right],t\right),$$

where *x*(*t*) ∈ ℝ^*d*^ is a time-varying vector representing the opinion of *d* individuals; $t\in [\overset{\vee }{t},\hat{t}]$ is the time; Vectors $\dot{x}\left(t\right),\ddot{x}\left(t\right),\overset{\mathrm{...}}{x}\left(t\right)$, and *x*^(*c*)^(*t*) correspond to the first, second, third, and *c*-th order time derivatives of *x*, respectively; The function **ϕ**:ℝ^*cd*^×ℝ → ℝ^*cd*^ is parameterized by a neural network with the parameter θ ∈ ℝ^*n*^. Note that ${\left[\dot{x}\left(t\right),\ddot{x}\left(t\right),\overset{\mathrm{...}}{x}\left(t\right),\ldots ,x^{\left(c\right)}\left(t\right)\right]}^{⊤}\in {\mathbb{R}}^{cd}$ is a concatenation of higher-order derivatives, where the superscript ^⊤^ means a transpose of a vector, and we call a HiDeS NODE with up to *c*-th order time derivatives in this concatenation as the HiDeS-*c* NODE. To enhance readability and avoid redundancy, we may omit “(*t*)” in certain contexts where the time dependency is understood and does not affect the meaning or clarity of the mathematical expressions.

Remark 1. One advantage of a HiDeS NODE is that it is able to model nonlinear interactions between higher-order derivatives and *x*. In practice, multiple higher-order derivatives and *x* can interact with each other. For example, there can be terms like $\dot{x}⊗\ddot{x}$ in the vector field, where ⊗ is the Hadamard product.

It can be seen that the standard NODE (Chen et al., 2018) is a HiDeS-1 NODE, and if we just focus on the formulation, SONODE (Norcliffe et al., 2020) can be regarded as a HiDeS-2 NODE. In fact, the HiDeS-2 NODE distinguishes itself from SONODE due to its unique training process.

### 3.2 Training of the HiDeS NODE

Existing variants of the NODE utilize the ground-truth value of *x*(*t*) as the label for training. Differently, the HiDeS NODE adopts the entire ${\left[x\left(t\right),\dot{x}\left(t\right),\ddot{x}\left(t\right),\ldots ,x^{\left(c-1\right)}\left(t\right)\right]}^{⊤}$ as the label (the model's prediction ${\left[\dot{x}\left(t\right),\ddot{x}\left(t\right),\overset{\mathrm{...}}{x}\left(t\right),\ldots ,x^{\left(c\right)}\left(t\right)\right]}^{⊤}$ is integrated first to get ${\left[x\left(t\right),\dot{x}\left(t\right),\ddot{x}\left(t\right),\ldots ,x^{\left(c-1\right)}\left(t\right)\right]}^{⊤}$). This approach is beneficial for predicting the future evolution of *x*(*t*). Training the entire ${\left[x\left(t\right),\dot{x}\left(t\right),\ddot{x}\left(t\right),\ldots ,x^{\left(c-1\right)}\left(t\right)\right]}^{⊤}$ gives accurate approximations of all these variables. Since the prediction of the next time step for the HiDeS NODE relies on the entire ${\left[x\left(t\right),\dot{x}\left(t\right),\ddot{x}\left(t\right),\ldots ,x^{\left(c-1\right)}\left(t\right)\right]}^{⊤}$, the training strategy of the HiDeS NODE leads to a better prediction performance compared to only training with the ground-truth value of *x*(*t*). When the model is predicting the next *x*(*t*), utilizing only the ground-truth value of *x*(*t*) as the label may lead to an inaccurate result because the basic information it relies on is inaccurate. The inclusion of the derivatives ensures that the model is sensitive to not just the position or condition at a given time but also to the trends and patterns of change, which are critical for forecasting. An explanation is illustrated in Figure 1.

### 3.3 Inexpressible trajectories of the HiDeS NODE

The superior expressive capability of the HiDeS NODE comes from two aspects.

The first is that lower-order NODEs have limitations in modeling trajectories that require the representation of higher-order dynamics. Consider a trajectory that requires an abrupt change in its acceleration (second derivative of *x*), which is not expressible in a first-order system but can be expressed in a second-order system ${\left[\dot{x}\left(t\right),\ddot{x}\left(t\right)\right]}^{⊤}=\phi_{\theta }\left({\left[x\left(t\right),\dot{x}\left(t\right)\right]}^{⊤},t\right)$. Similarly, trajectories requiring changes in the third derivative (jerk) are not expressible in a second-order system but can be captured in a third-order system, and so on. As a result, there exist trajectories that can not be expressed by ${\left[\dot{x}\left(t\right),\ddot{x}\left(t\right),\overset{\mathrm{...}}{x}\left(t\right),\ldots ,x^{\left(c\right)}\left(t\right)\right]}^{⊤}=\phi_{\theta }\left({\left[x\left(t\right),\dot{x}\left(t\right),\ddot{x}\left(t\right),\ldots ,x^{\left(c-1\right)}\left(t\right)\right]}^{⊤},t\right)$ but can be expressed by ${\left[\dot{x}\left(t\right),\ddot{x}\left(t\right),\overset{\mathrm{...}}{x}\left(t\right),\ldots ,x^{\left(c+1\right)}\left(t\right)\right]}^{⊤}=\phi_{\theta }\left({\left[x\left(t\right),\dot{x}\left(t\right),\ddot{x}\left(t\right),\ldots ,x^{\left(c\right)}\left(t\right)\right]}^{⊤},t\right)$.

The second origin of the superior expressive capability of the HiDeS NODE is that it alleviates the restriction that trajectories cannot cross. One major limitation of the standard NODE is that trajectories under different initial conditions cannot intersect, which constrains its expressive capability. In the following, we show how this constraint is able to be eliminated by the HiDeS NODE.

Theorem 1 (Inexpressible trajectories of a HiDeS-*c* NODE). Assume that the function $\phi_{\theta }\left(\omega ,t\right):{\mathbb{R}}^{cd}\times \mathbb{R}\to {\mathbb{R}}^{cd}$ with $t\in [\overset{\vee }{t},\hat{t}]$ is Lipschitz continuous w.r.t. ω ∈ ℝ^*cd*^. Consider a HiDeS-*c* NODE governed by Equation (2):


(2) $$\begin{array}{l} {\left[\dot{x}\left(t\right),\ddot{x}\left(t\right),\overset{\mathrm{...}}{x}\left(t\right),\ldots ,x^{\left(c\right)}\left(t\right)\right]}^{⊤}\\ \;=\phi_{\theta }\left({\left[x\left(t\right),\dot{x}\left(t\right),\ddot{x}\left(t\right),\ldots ,x^{\left(c-1\right)}\left(t\right)\right]}^{⊤},t\right),\; \end{array}$$

where *x*(*t*) ∈ ℝ^*d*^ is the state vector, $\phi_{\theta }:{\mathbb{R}}^{cd}\times \mathbb{R}\to {\mathbb{R}}^{cd}$ is a continuously differentiable function parameterized by neural network parameters **θ**. For any two initial conditions $\omega \left(\overset{\vee }{t}\right)={\left[x\left(\overset{\vee }{t}\right),\dot{x}\left(\overset{\vee }{t}\right),\ldots ,x^{\left(c-1\right)}\left(\overset{\vee }{t}\right)\right]}^{⊤}$ and $\tilde{\omega }\left(\overset{\vee }{t}\right)={\left[\tilde{x}\left(\overset{\vee }{t}\right),\dot{\tilde{x}}\left(\overset{\vee }{t}\right),\ldots ,{\tilde{x}}^{\left(c-1\right)}\left(\overset{\vee }{t}\right)\right]}^{⊤}$, trajectories that require ω(*t*) and $\tilde{\omega }\left(t\right)$ to cross over the interval $\left[\overset{\vee }{t},\hat{t}\right]$ are inexpressible by a HiDeS-*c* NODE.

*Proof*. Define the extended state vector **ω** ∈ ℝ^*cd*^ as $\omega \left(t\right)={\left[x\left(t\right),\dot{x}\left(t\right),\ddot{x}\left(t\right),\ldots ,x^{\left(c-1\right)}\left(t\right)\right]}^{⊤}$. The HiDeS-*c* NODE can be represented as Equation (3):


(3) $$\begin{array}{l} \dot{\omega }=\phi_{\theta }\left(\omega \left(t\right),t\right). \end{array}$$

Given that **ϕ**_θ_ is Lipschitz continuous w.r.t. **ω**, the Picard-Lindelöf theorem (Anil Kumar et al., 2022; Zhang et al., 2023) assures the existence of a unique solution **ω**(*t*) for a given initial condition **ω**(0). This uniqueness implies that for any two distinct initial conditions ($\omega (\overset{\vee }{t})$) and $\tilde{\omega }\left(\overset{\vee }{t}\right)$, the resulting trajectories **ω**(*t*) and $\tilde{\omega }\left(t\right)$ do not cross over $\left[\overset{\vee }{t},\hat{t}\right]$. As a result, trajectories that require **ω**(*t*) and $\tilde{\omega }\left(t\right)$ to cross over the interval $\left[\overset{\vee }{t},\hat{t}\right]$ are inexpressible by a HiDeS-*c* NODE. The proof is thus completed.          ⎕

Remark 2. From Theorem 1, it can be seen that as the order *c* increases, the degree of freedom for avoiding the crossing of *x*(*t*) and $\tilde{x}\left(t\right)$ increases. In practice, there are some trajectories cross in the *x*-*t* space or in the phase space [i.e., *x*-$\dot{x}$-$\ddot{x}$-…-*x*^(*c*−1)^-*t* space], so the HiDeS-*c* NODE provides a better capability to model these dynamics compared with the standard NODE (a HiDeS-1 NODE) (Chen et al., 2018) and other second-order NODEs (HiDeS-2 NODEs) (Norcliffe et al., 2020; Sander et al., 2021). An intuitive understanding is that additional dimensions provide new directions to make trajectories elude each other, which is illustrated in Figure 1. Consider two actual evolution trajectories under different initial conditions, x^*^(*t*) and ${\tilde{x}}^{*}\left(t\right)$, which may intersect at some time points if there are no restrictions. However, for *c* = 1, the trajectories *x*(*t*) and $\tilde{x}\left(t\right)$ generated by the standard NODE cannot intersect due to its nature of first-order ODEs. This limitation means they cannot accurately approximate x^*^(*t*) and ${\tilde{x}}^{*}\left(t\right)$ in cases where the actual trajectories intersect. Theorem 1 from our manuscript implies similar limitations for higher-order NODEs, but with increasing order *c*, the trajectories have more freedom, reducing the limitations.

### 3.4 The HiDeS NODE's utilization of historical information

Due to the introduction of higher-order derivatives, the HiDeS NODE implicitly uses historical state information for predicting the next state. The reason is that higher-order derivatives can be approximated by historical states. For example, the first derivative $\dot{x}$ at the *k*-th moment *t*_*k*_ can be approximated as $\dot{x}\left(t_{k}\right)\approx \left(x\left(t_{k}\right)-x\left(t_{k-1}\right)\right)/\Delta t$. Similarly, the second derivative $\ddot{x}$ can be approximated as Equation (4):


(4) $$\begin{array}{l} \ddot{x}\left(t_{k}\right)\approx \frac{\left(\frac{x\left(t_{k}\right)-x\left(t_{k-1}\right)}{\Delta t}\right)-\left(\frac{x\left(t_{k-1}\right)-x\left(t_{k-2}\right)}{\Delta t}\right)}{\Delta t}. \end{array}$$

Iteratively, we have Equation (5):


(5) $$\begin{array}{l} x^{\left(c\right)}(t_{k})\approx \sum_{i=0}^{c}{\left(-1\right)}^{i}\left({\;}_{i}^{c}\right)x(t_{k-i})/(\Delta t^{c}), \end{array}$$

where the binomial coefficient $\left({\;}_{i}^{c}\right)=c!/\left(i!\left(c-i\right)!\right)$ represents the combinatorial number of ways to choose *i* elements from a set of *c* elements. Consequently, $\phi_{\theta }\left({\left[x\left(t\right),\dot{x}\left(t\right),\ddot{x}\left(t\right),\ldots ,x^{\left(c-1\right)}\left(t\right)\right]}^{⊤},t\right)$ in the HiDeS NODE (Equation 1) can be approximated as a function of historical states as in Equation (6):


(6) $$\begin{array}{l} {\left[\dot{x}(t),\overset{..}{x}(t),\overset{...}{x}(t),\ldots ,x^{\left(c\right)}(t)\right]}^{⊤}\\ \;=\phi_{\theta }\left({\left[x(t),\dot{x}(t),\overset{..}{x}(t),\ldots ,x^{\left(c-1\right)}(t)\right]}^{⊤},t\right)\\ \;\approx \;\phi_{v}\left(x(t_{k}),\;x(t_{k-1}),\ldots ,\;x(t_{k-c}),t_{k}\right), \end{array}$$

where $\phi_{\vartheta }:{\mathbb{R}}^{d}\times {\mathbb{R}}^{d}\times \cdots \times \mathbb{R}\to {\mathbb{R}}^{cd}$ is a function parameterized by ϑ. The HiDeS NODE's utilization of historical information could enhance the prediction of the next state.

### 3.5 Implementation

**Figure 2:**
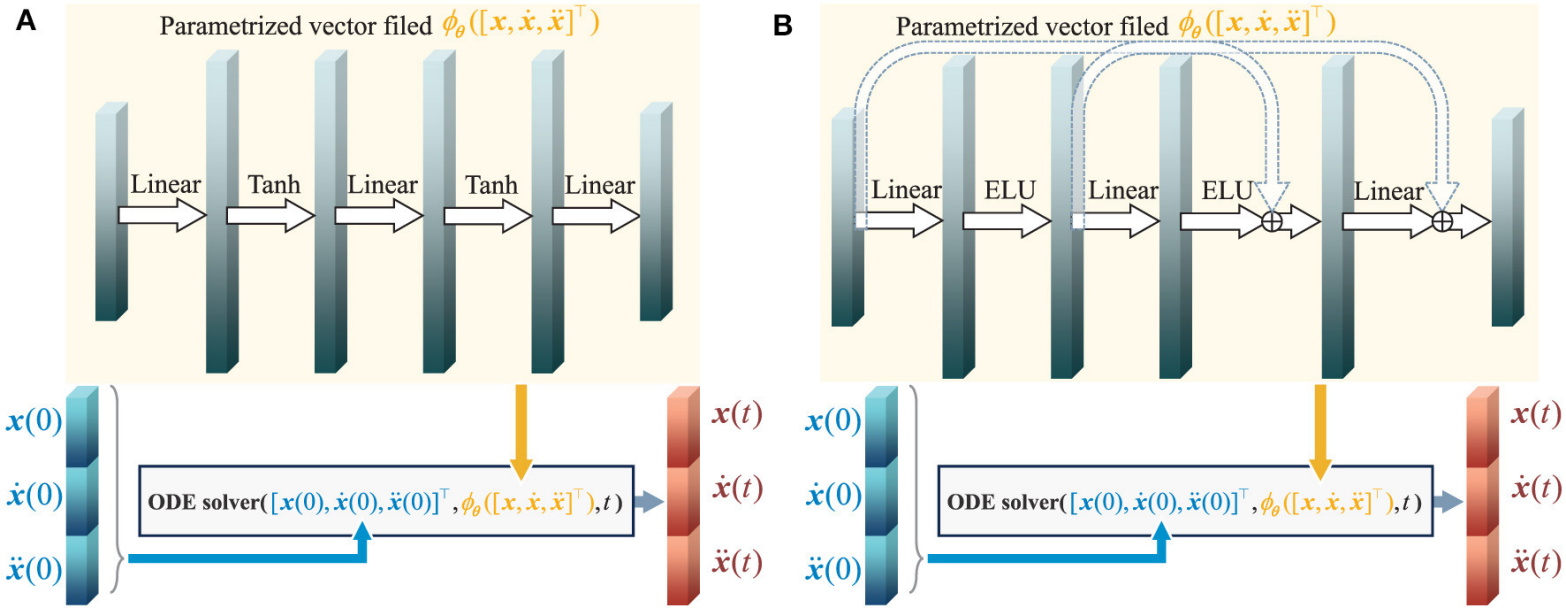
Structure of HiDeS-3 NODEs. **(A)** Structure of HiDeS-3 NODE for multi-robot systems. **(B)** Structure of HiDeS-3 NODE for opinion dynamics.

We provide Algorithm 1 to show the process of constructing, training, and using a HiDeS NODE. Besides, structures of HiDeS-3 NODEs for multi-robot systems and for opinion dynamics are shown in Figures 2A, B, respectively. In Figure 2, the inputs to the system are a concatenation of the initial state *x*(0), the initial velocity $\dot{x}\left(0\right)$, and the initial acceleration $\ddot{x}\left(0\right)$, which are fed into an ODE solver alongside the parametrized function $\phi_{\theta }\left({\left[x\left(t\right),\dot{x}\left(t\right),\ddot{x}\left(t\right)\right]}^{⊤}\right)$ to compute the state *x*(*t*), velocity $\dot{x}\left(t\right)$, and acceleration $\ddot{x}\left(t\right)$ at time *t*.

**Algorithm 1:**
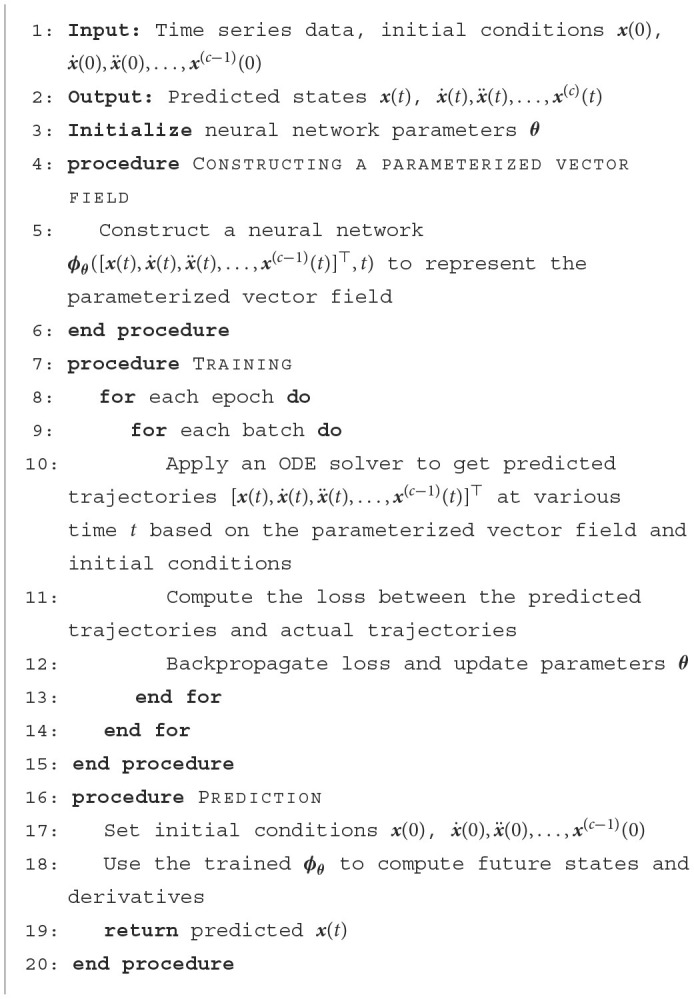
Algorithm of HiDeS NODE.

## 4 Results

In this section, we conduct experiments to evaluate the effectiveness of our models, HiDeS-2 NODE and HiDeS-3 NODE, by comparing them with baseline models [standard NODE (Chen et al., 2018) and SONODE (Norcliffe et al., 2020)] on two applications: multi-robot control and opinion dynamics. These baseline models have the same configurations in terms of network architecture, optimizer, epochs, and learning rate, ensuring a fair comparison. Notably, our models utilize higher-order derivatives as supervised signals, crucial for accurately capturing the intricate, nonlinear evolution of opinions over time. The implementation details of our experiments are as follows.

### 4.1 Experimental settings

#### 4.1.1 Settings for multi-robot-system control

In multi-robot-system control, each NODE block is composed of three fully connected layers, each succeeded by a Tanh activation function, as shown in Figure 2A. The NODE block undergoes forward propagation 200 times, evolving from *t* = 0 to *t* = 20, in order to develop a deep model. A weighted loss ℓ that emphasizes the trajectory's later stages is applied: $\ell_{\text{W}}=\overset{\hat{k}}{\underset{k=0}{\sum }}{\left(k/\hat{k}\right)}^{p}\ell \left(\omega \left(t_{k}\right),\omega^{*}\left(t_{k}\right)\right)$, where $\hat{k}$ is the total number of steps, *p*>0 is a scalar, ℓ(·, ·) is a loss function, and $\omega^{*}\left(t_{k}\right)$ is the ground truth of **ω**(*t*_*k*_). In simulations, *p* is taken as 4. All models are trained for 1,000 epochs using the Adam optimizer and a cosine annealing scheduler with a base learning rate of 0.01.

#### 4.1.2 Settings for opinion dynamics

In simulations of opinion dynamics, each block of NODEs consists of three fully connected layers, each followed by an exponential linear unit (ELU) activation function, as shown in Figure 2B. The block of NODEs loops in the forward propagation 50 times from *t* = 0 to *t* = 5 to form a deep model. To enhance the training stability, we incorporate residual connections. The optimizer employed is NAdam (Dozat, 2016; Li et al., 2022), with an initial learning rate of 0.01, modulated using a cosine annealing learning rate scheduler (Jin et al., 2022). We train the models over 1,000 and 2,000 epochs, respectively. The loss function is the mean square error between the predicted and actual $x\left(t\right),\dot{x}\left(t\right),\ddot{x}\left(t\right),\ldots ,x^{\left(c-1\right)}\left(t\right)$. Given the higher dimensions of HiDeS NODEs and SONODEs compared to standard NODEs, we introduce an auxiliary loss to ensure a fair comparison. The auxiliary loss is evaluated based on predicted and actual *x*(*t*), and it is only used for comparisons rather than for training purposes.

### 4.2 Target chasing of multi-robot system

In this section, the NODE models are used to control a multi-robot system to chase a moving target. The target's trajectory is described by *x*_1_ = 0.5*t* and *x*_2_ = 2sin(0.5*t*+2). During the training phase, this trajectory serves as the ground truth to minimize the total distance between the robots and the target. In the test phase, the target location is unknown. The transition from training to test simulates a scenario in tracking processes where, despite initially having knowledge of the target location, the information regarding the target's position is lost from a certain moment onward.

As tracking problems in reality often occur under finite energy consumption, we introduce an inequality constraint on the total energy consumption of all agents: *e* ≤ *e*_max_, where *e* represents the energy and *e*_max_ is the predetermined energy ceiling. This constraint is implemented during training through a regularization term as in Equation (7):


(7) $$\begin{array}{l} \overset{\vee }{\ell }=\ell \left(\omega ,\omega^{*}\right)+\max\left\{e_{\max},e\right\}. \end{array}$$

During training, if *e*>*e*_max_, the term max{*e*_max_, *e*} encourages a reduction in *e*; If *e* ≤ *e*_max_, then this term does not affect *e*. Since the tracking occurs on a horizontal plane, potential energy is not considered; thus, $e=\overset{r}{\underset{i}{\sum }}m_{i}v_{i}^{2}/2$, where *r* is the total number of robots. In the simulations, we set each robot's mass as equal, with the total mass being 2 kg (therefore, $e=\overset{r}{\underset{i}{\sum }}v_{i}^{2}=\overset{d}{\underset{j=0}{\sum }}\overset{r}{\underset{i=0}{\sum }}{ẋ}_{ij}^{2}$), and set *e*_max_ = 5,000 J.

#### 4.2.1 Chasing trajectories with given target trajectory

**Figure 3:**
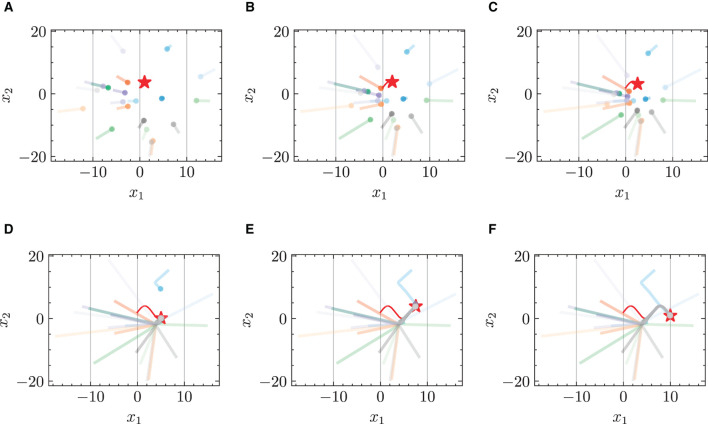
Trajectories of multiple robots using standard NODE in target-chasing task without energy constraint. This figure depicts the paths of multiple robots (indicated by dots) as they follow a dynamically moving target (denoted by stars) after 1,000 epochs of training. **(A)** *t* = 2 s. **(B)** *t* = 4 s. **(C)** *t* = 5 s. **(D)** *t* = 10 s. **(E)** *t* = 15 s. **(F)** *t* = 20 s.

**Figure 4:**
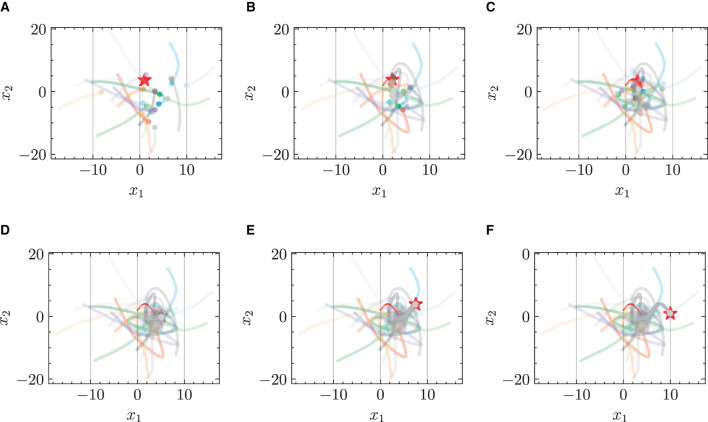
Trajectories of multiple robots using HiDeS-3 NODE (ours) in target-chasing task without energy constraint. This figure depicts the paths of multiple robots (indicated by dots) as they follow a dynamically moving target (denoted by stars) after 1,000 epochs of training. **(A)** *t* = 2 s. **(B)** *t* = 4 s. **(C)** *t* = 5 s. **(D)** *t* = 10 s. **(E)** *t* = 15 s. **(F)** *t* = 20 s.

**Figure 5:**
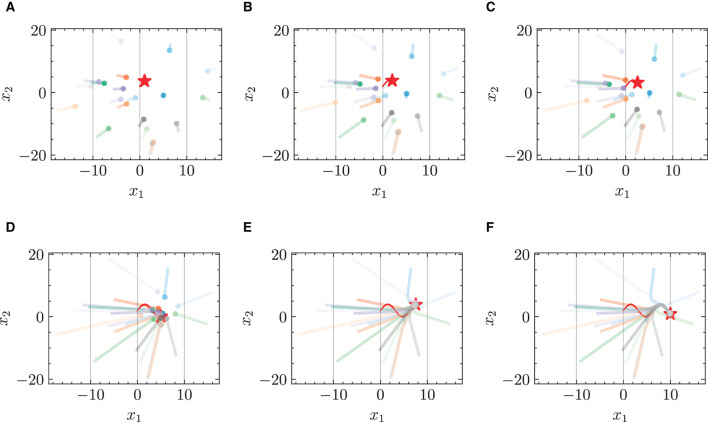
Trajectories of multiple robots using standard NODE in target-chasing task with energy constraint. This figure depicts the paths of multiple robots (indicated by dots) as they follow a dynamically moving target (denoted by stars) after 1,000 epochs of training. **(A)** *t* = 2 s. **(B)** *t* = 4 s. **(C)** *t* = 5 s. **(D)** *t* = 10 s. **(E)** *t* = 15 s. **(F)** *t* = 20 s.

**Figure 6:**
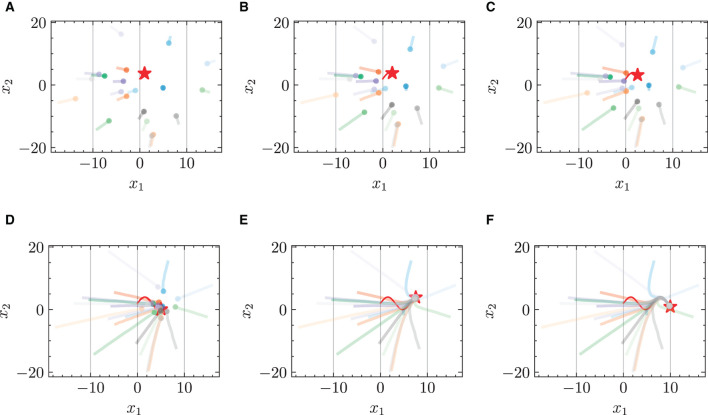
Trajectories of multiple robots using HiDeS-3 NODE (ours) in target-chasing task with energy constraint. This figure depicts the paths of multiple robots (indicated by dots) as they follow a dynamically moving target (denoted by stars) after 1,000 epochs of training. **(A)** *t* = 2 s. **(B)** *t* = 4 s. **(C)** *t* = 5 s. **(D)** *t* = 10 s. **(E)** *t* = 15 s. **(F)** *t* = 20 s.

Figures 3 and 4 respectively illustrate the trajectories of multiple robots and a chased target at different moments in time without energy constraints. By comparing Figures 3A and 4A, it can be observed that the chasing speed of the proposed HiDeS-3 NODE is significantly faster than the standard NODE. At the end of the tracking phase (e.g., at *t* = 20 s), both the standard NODE and HiDeS-3 NODE successfully reach the target. Examination of the various subfigures in Figure 4 reveals that the trajectories of the HiDeS-3 NODE exhibit typical characteristics of high-order dynamic systems similar to those seen with higher-order optimizers (Su et al., 2016; An et al., 2018) and proportional-integral-derivative (PID) controllers (Huba et al., 2023), such as rapid convergence and overshooting. This is attributed to HiDeS-3 NODE being a high-order dynamic system, as demonstrated by Equation 1. Figures 5 and 6 present a superficially similar performance between the standard NODE and the HiDeS NODE when subject to energy constraints. However, a distinct contrast emerges during the subsequent testing phase, which operates without a predefined target position.

#### 4.2.2 Predicted trajectories without given target position

**Figure 7:**
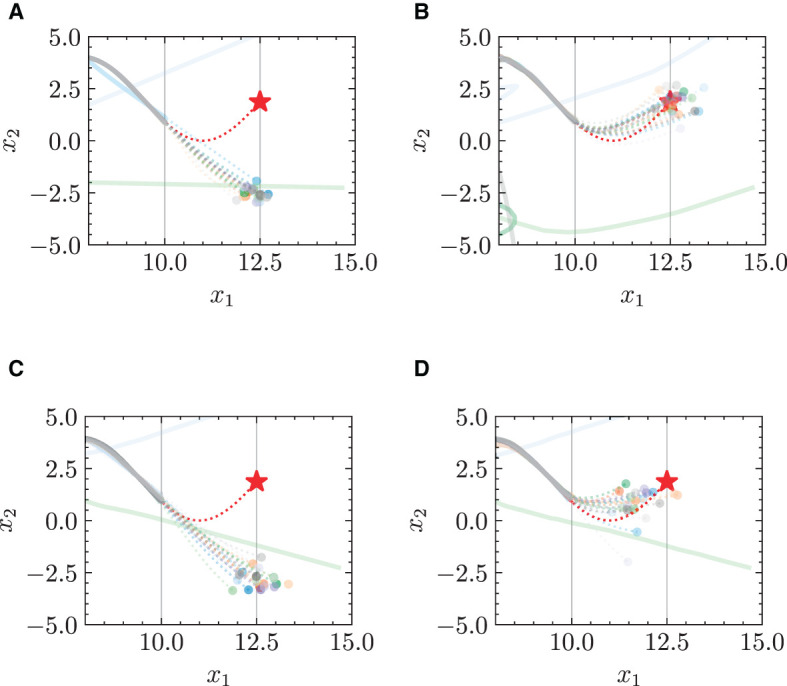
Trajectories of multiple robots in target-chasing task after losing information of target. This figure depicts the paths of multiple robots (indicated by dots) as they follow a dynamically moving target (denoted by stars). Solid curves denote training phase (trajectory of target is known), and dashed curves denote test phase (trajectory of target is unknown). **(A)** Standard NODE; without energy constraint. **(B)** HiDeS-3 NODE (ours); without energy constraint. **(C)** Standard NODE; with energy constraint. **(D)** HiDeS-3 NODE (ours); with energy constraint.

In the test phase, the target position is not given to the model. Figure 7 presents a comparison of the predicted trajectories of multi-robots in a target-chasing scenario where the target position is not provided. The comparison is between the trajectories generated by the standard NODE and the HiDeS NODE, with and without the imposition of energy constraints. The figure clearly demonstrates that the HiDeS NODE offers superior performance over the standard NODE. Specifically, the trajectories predicted by the standard NODE show significant deviations from the target (Figures 7A, C). In contrast, those predicted by the HiDeS NODE closely align with the target's trajectory. Although the HiDeS NODE with energy constraints shows slight deviations due to restricted velocity, it still significantly outperforms the standard NODE (Figures 7B, D). This suggests that the advantages of the HiDeS NODE may not solely be attributed to increased velocity but could also derive from additional information, such as curvature, which is inferred by the high-order supervised signals, as illustrated in the middle of Figure 1.

#### 4.2.3 Energy cost

**Figure 8:**
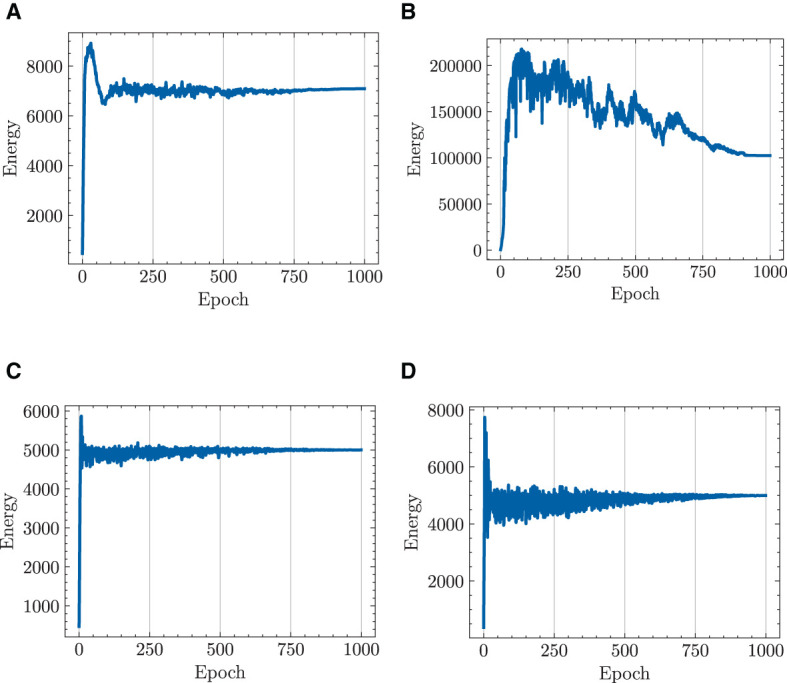
Total energy cost of multiple robots in target-chasing task during 1,000 epochs of training. Energy constraint is set such that total energy cost ≤5,000 J. **(A)** standard NODE; without energy constraint. **(B)** HiDeS-3 NODE (ours); without energy constraint. **(C)** Standard NODE; with energy constraint. **(D)** HiDeS-3 NODE (ours); with energy constraint.

Figure 8 draws a parallel of the total energy expenditure of multiple robots engaged in a target-chasing task, contrasting the standard NODE with the HiDeS NODE. The figure shows that the implementation of energy constraints has a significant impact. Without energy constraints, both the standard NODE and the HiDeS NODE incur substantial energy costs after training, reaching up to 7,000 and 100,000 J, respectively (Figures 8A,B). However, with the application of energy constraints, both NODEs manage to keep the energy expenditure no more than 5,000 J after 1,000 epochs of training (Figures 8C, D).

### 4.3 Modeling and predicting of opinion dynamics

In this section, simulations of NODEs on modeling and predicting opinion dynamics are conducted.

#### 4.3.1 One dimension, multiple initial conditions

**Figure 9:**
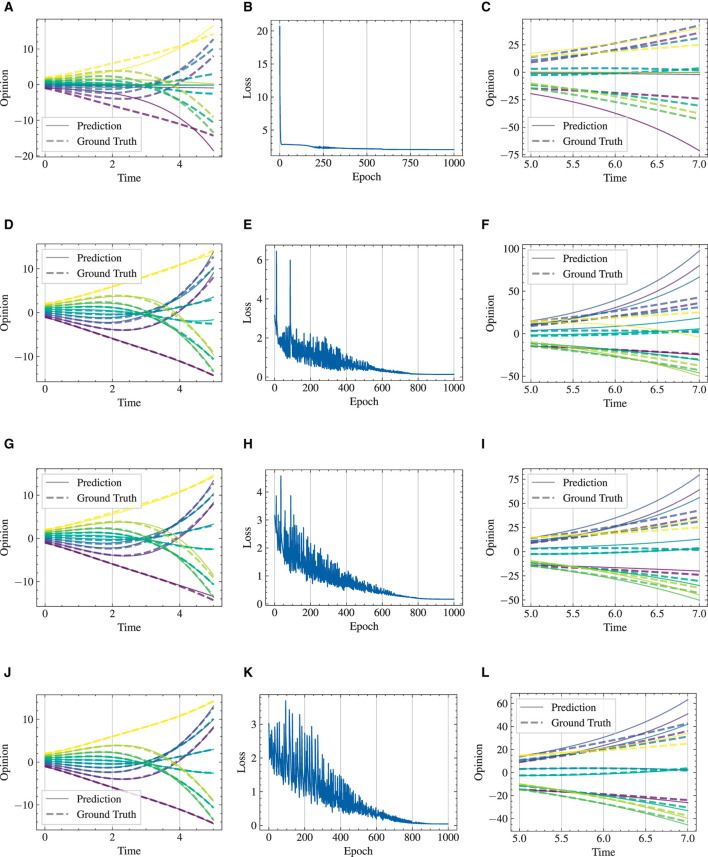
Visualization of learning results with standard NODE, SONODE, HiDeS-2 NODE, and HiDeS-3 NODE. All NODEs are trained for 1,000 epochs. **(A)** Standard NODE; opinion evolution; training. **(B)** Training loss w.r.t. epoch. **(C)** Standard NODE; opinion evolution; test. **(D)** SONODE; Opinion evolution; training. **(E)** Training loss w.r.t. epoch. **(F)** SONODE; opinion evolution; test. **(G)** HiDeS-2 NODE; opinion evolution; training. **(H)** Training loss w.r.t. epoch. **(I)** HiDeS-2 NODE; opinion evolution; test. **(J)** HiDeS-3 NODE; opinion evolution; training. **(K)** Training loss w.r.t. epoch. **(L)** HiDeS-3 NODE; opinion evolution; test.

**Figure 10:**
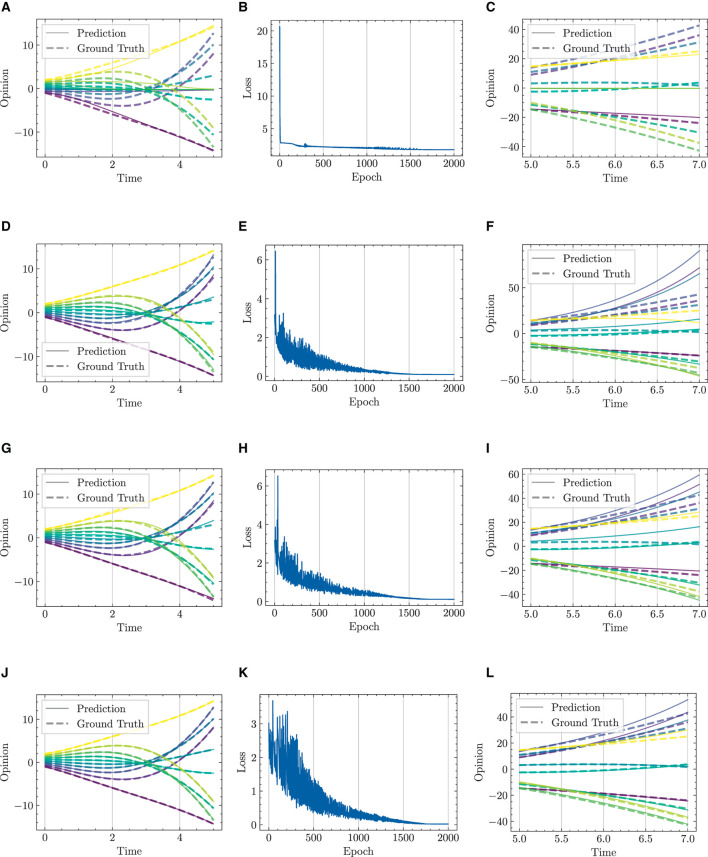
Visualization of learning results with standard NODE, SONODE, HiDeS-2 NODE, and HiDeS-3 NODE. All NODEs are trained for 2,000 epochs. **(A)** Standard NODE; opinion evolution; training. **(B)** Training loss w.r.t. epoch. **(C)** Standard NODE; opinion evolution; test. **(D)** SONODE; Opinion evolution; training. **(E)** Training loss w.r.t. epoch. **(F)** SONODE; opinion evolution; test. **(G)** HiDeS-2 NODE; opinion evolution; training. **(H)** Training loss w.r.t. epoch. **(I)** HiDeS-2 NODE; opinion evolution; test. **(J)** HiDeS-3 NODE; opinion evolution; training. **(K)** Training loss w.r.t. epoch. **(L)** HiDeS-3 NODE; opinion evolution; test.

We respectively present experimental results conducted over 1,000 and 2,000 epochs in Figures 9, 10, and complemented by Table 3. The results indicate that our HiDeS NODEs surpass the standard NODE and SONODE in capturing the subtleties of opinion dynamics. Note that the SONODE and HiDeS-2 have the same hidden layer dimensions. This superiority is evident from the more accurate approximations of actual opinion dynamics during both training and testing phases (Figures 9 and 10) and lower auxiliary losses in consistent iterations (Table 3). For instance, Figure 9 shows that the prediction curves of standard NODE diverge from the ground truth traces in both training and testing since the trajectories from different initial conditions can not cross. While SONODE performs better than standard NODE in the learning stage, its predictive ability remains inferior in the testing phase. In contrast, our models' prediction curves closely align with the ground truth. Extending the epochs to 2,000 shows that both models perform better in the learning stage than under 1,000 epochs, with our models demonstrating remarkable superiority in the testing phase. This empirical observation aligns with our theoretical analyses that using higher-order derivatives as supervised signals enhances the predictive capacity. Furthermore, as Table 3 shows, the proposed HiDeS-3 NODE exhibits significantly lower training and testing losses compared to the baseline models. Although the HiDeS-2 NODE exhibits a slightly higher training loss than SONODE, its test loss is substantially lower, indicating superior generalization ability, a key goal of neural networks.

**Table 3:** Comparisons of training and test losses among SOTA NODEs and HiDeS NODE.

**# Epochs**	**Loss**	**Standard NODE (Chen et al., 2018)**	**SONODE (Norcliffe et al., 2020)**	**HiDeS-2 NODE (ours)**	**HiDeS-3 NODE (ours)**
1,000	Training	2.05	0.13	0.17	0.04
	Test	17.50	6.97	4.42	2.03
2,000	Training	1.78	0.09	0.12	0.02
	Test	14.70	5.21	2.63	1.08

#### 4.3.2 Multiple dimension, one initial condition

**Figure 11:**
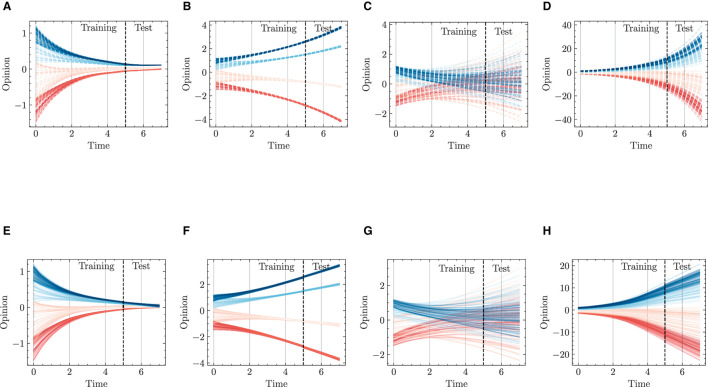
Opinion dynamics modeling and predicting using HiDeS-3 NODE for different consensus and dissensus scenarios. **(A)** Intra-group consensus and inter-group consensus, ground truth. **(B)** Intra-group consensus and inter-group dissensus, ground truth. **(C)** Intra-group dissensus and inter-group consensus, ground truth. **(D)** Intra-group dissensus and inter-group dissensus, ground truth. **(E)** Intra-group consensus and inter-group consensus, predicted by HiDeS-3 NODE. **(F)** Intra-group consensus and inter-group dissensus, predicted by HiDeS-3 NODE. **(G)** Intra-group dissensus and inter-group consensus, predicted by HiDeS-3 NODE. **(H)** Intra-group dissensus and inter-group dissensus, predicted by HiDeS-3 NODE.

Figure 11 presents modeling and predicting results on diverse types of individuals' intra- and inter-group interactions for opinion dynamics. The top row (Figures 11A–D) represents ground truth values, while the bottom row (Figures 11E, F) shows predictions generated by the HiDeS-3 NODE. Each subfigure illustrates different combinations of consensus and dissensus within and between groups, highlighting the model's performance in capturing extensive opinion dynamics.

#### 4.3.3 Training dynamics

**Figure 12:**
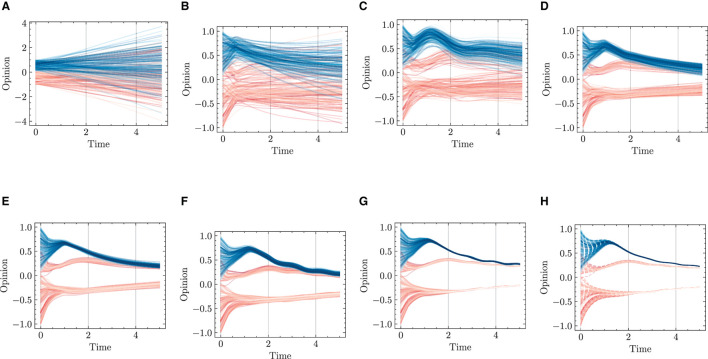
Opinion dynamics learning results during 2,000-epoch training. **(A)** Epoch = 1. **(B)** Epoch = 100. **(C)** Epoch = 200. **(D)** Epoch = 500. **(E)** Epoch = 1,000. **(F)** Epoch = 1,100. **(G)** Epoch = 2,000. **(H)** Ground truth.

The learning results of opinion evolution are shown in Figure 12 with the increase of epochs during a 2,000-epoch training. It is clear that the learning results get more accurate and fine-grained with the increasing epochs. Specifically, the predicted results show significant deviation from the ground truth under 1–200 epochs (Figures 12B, C), while the more granular learning results are presented with the increase of epochs (Figures 12D–H), achieving more accurate predictions for opinion evolution.

## 5 Discussion

This paper has proposed the HiDeS NODE, a higher-order-derivative-supervised NODE, as a novel approach for modeling and predicting complex dynamics in multi-robot systems and opinion dynamics. This framework excels in capturing interactions among higher-order derivatives and the state vector, significantly enhancing modeling precision over existing NODE methodologies. The introduction of higher-order derivatives as supervised signals in the HiDeS NODE brings a superior predicting ability. Applications of the HiDeS NODE in multi-robot systems and opinion dynamics have demonstrated its effectiveness. To our knowledge, this is the first initiative that introduces NODEs into multi-robot systems and opinion dynamics. Applying the HiDeS NODE to these fields opens new avenues for broader applications in various intricate and dynamic systems.

The broader impact of the HiDeS NODE extends into numerous fields where dynamic systems play a crucial role. For instance, it has the potential to offer refined predictions of climate change effects or pollution dispersion. In healthcare, the HiDeS NODE could lead to breakthroughs in understanding the dynamics of disease spread or patient response to treatments, enabling personalized medicine. The adaptability and advanced modeling capabilities of the HiDeS NODE position it as a versatile tool capable of addressing complex problems across various domains.

Despite its potential, the HiDeS NODE faces limitations such as computational demands, particularly as the order of derivatives increases, making real-time applications challenging. The model's accuracy is heavily reliant on the quality and quantity of data, which can be a significant constraint in environments where data is sparse or noisy. Addressing these challenges will be essential for the HiDeS NODE's successful application across different fields.

A valuable future direction is to utilize real-world data to validate our model's performance in practical scenarios. Additionally, enhancing the robustness of the HiDeS NODE to noisy data presents a promising direction for future research.

## Data availability statement

The original contributions presented in the study are included in the article/supplementary material, further inquiries can be directed to the corresponding author.

## Author contributions

MLi: Conceptualization, Formal analysis, Methodology, Software, Writing – original draft. WB: Data curation, Funding acquisition, Resources, Supervision, Validation, Visualization, Writing – review & editing. LC: Funding acquisition, Project administration, Resources, Software, Validation, Visualization, Writing – review & editing. MLiu: Data curation, Formal analysis, Investigation, Resources, Supervision, Validation, Writing – review & editing.
